# Endemic Hantavirus in Field Voles, Northern England

**DOI:** 10.3201/eid2306.161607

**Published:** 2017-06

**Authors:** Anna G. Thomason, Michael Begon, Janette E. Bradley, Steve Paterson, Joseph A. Jackson

**Affiliations:** University of Salford, Salford, UK (A.G. Thomason, J.A. Jackson);; University of Liverpool, Liverpool, UK (M. Begon, S. Paterson);; University of Nottingham, Nottingham, UK (J.E. Bradley)

**Keywords:** *Microtus*, *Microtus agrestis*, field vole, hantavirus, Tatenale virus, United Kingdom, England, viruses, zoonoses

## Abstract

We report a PCR survey of hantavirus infection in an extensive field vole (*Microtus agrestis*) population present in the Kielder Forest, northern England. A Tatenale virus–like lineage was frequently detected (≈17% prevalence) in liver tissue. Lineages genetically similar to Tatenale virus are likely to be endemic in northern England.

Recently a new vole-associated hantavirus (Tatenale virus) was discovered in northern England (*1*), but only from an individual *Microtus agrestis* field vole*.* Previously only hantaviruses from murine-associated lineages (Seoul virus [SEOV] and SEOV-like viruses) had been reported in the United Kingdom, despite the abundance of potential vole hosts in the mainland United Kingdom and the endemicity of vole-associated hantavirus lineages (Puumala virus [PUUV] and Tula virus) in mainland Europe (*2*). Here we present data suggesting that the Tatenale virus lineage is endemic in northern England.

European hantaviruses are of public health significance because they are a causative agent of hemorrhagic fever with renal syndrome (HFRS). In the United Kingdom, HFRS cases have primarily been attributed to SEOV-like viruses on the basis of serologic tests. SEOV antibodies have been detected in both humans and Norway rats (*Rattus norvegicus*) in Northern Ireland and Yorkshire (*3*,*4*), and seropositivity in humans correlates with domestic or occupational exposure to rats (*3*,*5*). However, in the United Kingdom, HFRS cases with serologic cross-reactivity to PUUV (*3*), which might share antigenic determinants with Tatenale virus, have occurred.

To investigate the endemicity of hantavirus in field voles in the United Kingdom, we surveyed the extensive field vole population in the Kielder Forest, Northumberland (≈230 km distant from the locality where Tatenale virus was discovered). All sampled sites were grassy, clear-cut areas (adjacent to forest stands) where field voles were prevalent. Fieldwork was approved by the University of Liverpool Animal Welfare Committee and conducted subject to UK home office project license PPL 70_8210. Following the capture and processing of animals as previously described (*6*), we extracted viral RNA from 48 livers using a QIAamp Viral RNA Mini Kit (QIAGEN, Manchester, UK) and converted to cDNA using a High-Capacity RNA-to-cDNA Kit (Applied Biosystems, Warrington, UK). Hantaviruses were detected by PCR amplification of a fragment of the genomic L segment encoding RNA polymerase, following the strategy outlined by Klempa et al. (*7*).

PCR-positive results were recorded for 16.7% of voles (8/48) in total; positive voles were recorded at 3 of the 5 sampled sites and throughout the survey period, March–September 2015 (Figure, panel A; Technical Appendix Table). Three positive samples from different individual voles were sequenced (in both directions from independent replicate PCRs) by Sanger sequencing (Source BioScience, Rochdale, UK). The 380-bp sequence was identical in all 3 positive vole samples, with the exception of a single nucleotide polymorphism at position 145 (adenine, 2 voles; guanine, 1 vole; GenBank accession nos. KY751731, KY751732). The recovered sequences were similar to but divergent from Tatenale virus (86.0%–86.3% identity at the nucleotide level and 95.9%–96.7% identity at the amino acid level; Technical Appendix Figures 1, 2). Phylogenetic analysis of the L segment demonstrated this level of divergence was comparable to the divergence among many western European lineages of PUUV (Figure, panel B; Technical Appendix Figure 3).

**Figure:**
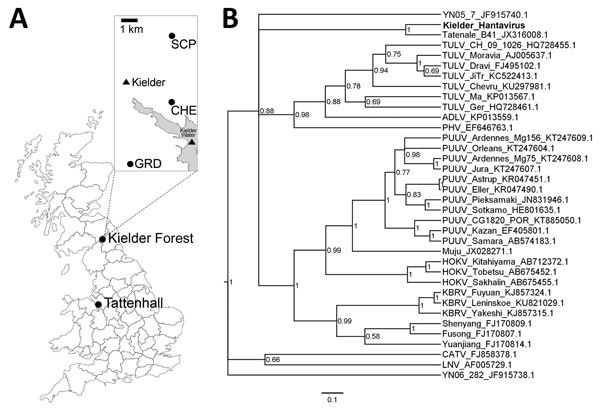
Investigation of Tatenale virus–like hantavirus lineages in the United Kingdom. A) Locations on the UK mainland where Tatenale virus–like hantavirus lineages have been found: Tattenhall, Cheshire, the site where Tatenale virus was discovered (*1*); and Kielder Forest, Northumberland. Kielder Forest sample sites are indicated in the inset (Geordies Knowe [GRD]: 55°11′1.61′′N, 2°35′3.05′′W; Cheese Sike [CHE]: 55°13′8.39′′N, 2°32′26.50′′W; Scaup [SCP]: 55°15′44.18′′N, 2°32′41.05′′W). B) Bayesian phylogenetic tree for the hantavirus genomic large segment (318-bp fragment with no missing data), showing relationships among Tatenale virus-like lineages and other relevant lineages. Bold represents the Tatenale virus–like lineage found in this study; either sequence reported here produces the same tree. Phylogenetic analysis was conducted by using a general time reversible plus gamma plus invariant sites model within MrBayes (*8*) software using Markov chain Monte Carlo chain lengths of 1 million and a strict clock. We estimated substitution models using MrModelTestV2 (*9*). Sequences are represented by the taxonomic names, strain (if >1 is included), and GenBank accession numbers. The tree is drawn to scale with node values representing the posterior probabilities. Scale bar represents nucleotide substitutions per site. ADLV, Adler virus; CATV, Catacamas virus; HOKV, Hokkaido virus; KBRV, Khabarovsk virus; LNV, Laguna Negra virus; PHV, Prospect Hill virus; PUUV, Puumala virus; TULV, Tula virus.

Taken together with the original report, these data are sufficient to suggest that Tatenale-like hantavirus lineages are widespread and common in northern England. Furthermore, the considerable sequence divergence between samples in Cheshire and Northumberland is consistent with a long-standing endemicity in northern England. Given that PUUV has never been recorded in the United Kingdom (*2*,*10*), the possibility should be considered that a Tatenale-like virus could have been responsible for some of the HFRS cases that have occurred here. More studies are needed to confirm whether other common rodents in the United Kingdom are hosts for this virus and to further characterize its phyletic relationships and zoonotic potential. Cross-reactivity of the sera from Tatenale-like virus–infected individuals to antigens of other relevant hantaviruses should be determined to inform future serologic surveys and the diagnosis of human HFRS cases.

Technical Appendix Number of hantavirus-infected voles found in Kielder Forest per trapping site, nucleotide and amino acid alignment of the L segment, and phylogenetic analysis of the L segment.
