# Supplementary material for: Endemic Hantavirus in Field Voles, Northern England
Source: Emerg Infect Dis. 2017 Jun;23(6):1033–5. doi: 10.3201/eid2306.161607 (PMC5443433; doi:10.3201/eid2306.161607)
Supplement: Technical Appendix — Number of hantavirus-infected voles found in Kielder Forest per trapping site, nucleotide and amino acid alignment of the L segment, and phylogenetic analysis of the L segment. [file 16-1607-Techapp-s1.pdf]

Article DOI: https://dx.doi.org/10.3201/eid2306.161607

# Endemic Hantavirus in Field Voles, Northern England

## Technical Appendix

**Technical Appendix Table.** Hantavirus detection by PCR of the viral genomic L segment in 48 voles, by sex and trapping site, northern England, March–September, 2015*

| | Sex | | Trapping sites | | | | |
|---|---|---|---|---|---|---|---|
| Category | M | F | SCP | CHE | GRD | COL | RAV |
| No. voles infected | 6 | 2 | 1 | 4 | 3 | 0 | 0 |
| Total voles | 34 | 14 | 12 | 18 | 12 | 2 | 4 |
| % infected | 17.65 | 14.29 | 8.33 | 22.22 | 25.00 | 0 | 0 |

*CHE, Cheese Sike (55°13′8.39″N, 2°32′26.50″W); COL, Coal Sike (55°14′08.35″N, 2°36′16.67″W); F, female; GRD, Geordies Knowe (55°11′1.61″N, 2°35′3.05″W); M, male; RAV, Ravenshill (55°14′30.55″N, 2°35′30.41″W); SCP, Scaup (55°15′44.18″N, 2° 32′41.05″W).

```
Kielder            1 TGGTCTCCAGGAGATAACTCTGCAAAGTTTAGAAGATTTACTCAGGCTCT     50
                     |||||||||||||||||.||.|||||||||||||..||.|||||||||||
Tatenale           1 TGGTCTCCAGGAGATAATTCGGCAAAGTTTAGAAAGTTCACTCAGGCTCT     50

Kielder           51 GTATGACGGCCTAAGGGATGATAAGTTAAAAAATTGTGTGGTGGATGCGT    100
                     ||||||.||..||.|.||||||||.||||||||||||||.||.|||||.|
Tatenale          51 GTATGATGGTTTACGAGATGATAAATTAAAAAATTGTGTAGTAGATGCAT    100

Kielder          101 TAAGAAACATTTATGAAACAGAATTTTTTATGTCTCGAGAGCTACATCGA    150
                     |||||||.||||||||||||||.||.||||||||.||..|..|.|||||.
Tatenale         101 TAAGAAATATTTATGAAACAGAGTTCTTTATGTCCCGGAAATTGCATCGG    150

Kielder          151 TATATAGATGGAATGGAAGATTTATCTGAAAATGTGGAAGATTTTCTATC    200
                     |||||.||.||.|||||||||||||||||||||||.|||||||||.|.||
Tatenale         151 TATATTGACGGGATGGAAGATTTATCTGAAAATGTAGAAGATTTTTTGTC    200

Kielder          201 ATTTTTTCCTAATAATGTCTCCGCAATGATCAAAGGTAATTGGTTGCAAG    250
                     ||||||.||.|||||||||||.||||||||.||||||||.|||.|.||.|
Tatenale         201 ATTTTTCCCCAATAATGTCTCTGCAATGATTAAAGGTAACTGGCTACAGG    250

Kielder          251 GTAATTTAAATAAGTGCTCATCATTATTTGGTGCTGCAGTATCCCTGTTA    300
                     ||||||||||||||||.|||||||||||||||||||||||.|||||||||
Tatenale         251 GTAATTTAAATAAGTGTTCATCATTATTTGGTGCTGCAGTGTCCCTGTTA    300

Kielder          301 TTTAAGAGGGTATGGACAAATTTATTCCCTGAATTAGACTGTTTCTTTGA    350
                     ||.|||||.||.||||||||||||||||||||.|||||.|||||||||||
Tatenale         301 TTCAAGAGAGTGTGGACAAATTTATTCCCTGAGTTAGATTGTTTCTTTGA    350

Kielder          351 ATTTGCCCACCACTCAGACGA    371
                     .|||||.||||.|.|...|||
Tatenale         351 GTTTGCACACCCCGCTCTCGA    371
```

**Technical Appendix Figure 1.** Pairwise nucleotide alignment of the genomic L segment sequence of the hantavirus found in Kielder Forest (145>A variant, GenBank accession no. KY751731) and Tatenale virus (GenBank accession no. JX316008.1). Sequences were aligned by using EMBOSS (*12*) on default settings. Alignment shows nucleotide similarity to be 86%.

```
Kielder           1 WSPGDNSAKFRRFTQALYDGLRDDKLKNCVVDALRNIYETEFFMSRELHR     50
                    |||||||||||:||||||||||||||||||||||||||||||||||:|||
Tatenale          1 WSPGDNSAKFRKFTQALYDGLRDDKLKNCVVDALRNIYETEFFMSRKLHR     50

Kielder          51 YIDGMEDLSENVEDFLSFFPNNVSAMIKGNWLQGNLNKCSSLFGAAVSLL    100
                    ||||||||||||||||||||||||||||||||||||||||||||||||||
Tatenale         51 YIDGMEDLSENVEDFLSFFPNNVSAMIKGNWLQGNLNKCSSLFGAAVSLL    100

Kielder         101 FKRVWTNLFPELDCFFEFAHHSD    123
                    ||||||||||||||||||||.:.
Tatenale        101 FKRVWTNLFPELDCFFEFAHPAL    123
```

**Technical Appendix Figure 2.** Pairwise amino acid alignment of the L segment of the Kielder Forest hantavirus with that of the corresponding Tatenale virus. The Kielder Forest hantavirus 145>A variant (GenBank accession no. KY751731) and Tatenale virus (GenBank accession no. JX316008.1) nucleotide sequences were translated with MEGA7 (*11*) and aligned with EMBOSS needle (*12*). Alignment shows amino acid identity at 95.9% and similarity at 98.4%.

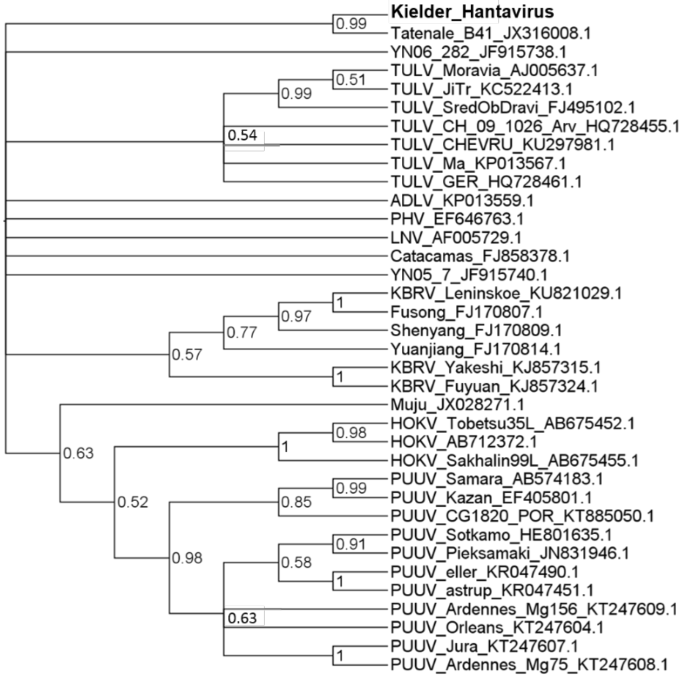


**Technical Appendix Figure 3.** A bootstrap consensus phylogenetic tree of the hantavirus genomic L segment constructed by using the maximum likelihood method based on the Tamura 3-parameter (G+I) model. Tree shows relationships among Tatenale virus-like lineages and other relevant lineages. Molecular analysis and model prediction were conducted using MEGA7 (*11*) with a bootstrap consensus inferred from 10,000 replicates. Node values represent the bootstrap values. Sequences are represented

by the taxonomic names, strain (if >1 is included), and GenBank accession numbers. Kielder Hantavirus (in bold) represents the 145>A variant found in this study. ADLV, Adler virus; CATV, Catacamas virus; HOKV, Hokkaido virus; KBRV, Khabarovsk virus; LNV, Laguna Negra virus; PHV, Prospect Hill virus; PUUV, Puumala virus; TULV, Tula virus.

## References


11. Kumar S, Stecher G, Tamura K. MEGA7: Molecular Evolutionary Genetics Analysis version 7.0 for bigger datasets. Mol Biol Evol. 2016;33:1870–4. PubMed http://dx.doi.org/10.1093/molbev/msw054

12. Rice P, Longden I, Bleasby A. EMBOSS: the European Molecular Biology Open Software Suite. Trends Genet. 2000;16:276–7. PubMed http://dx.doi.org/10.1016/S0168-9525(00)02024-2
